# Evaluation of Diagnosis, Cross‐Reactivity, and Risk Factors in Pediatric Patients With Macrolide Allergy

**DOI:** 10.1002/clt2.70096

**Published:** 2025-08-26

**Authors:** Güler Yıldırım, Merve Karaca Şahin, Nilay Çalışkan, Hamit Boloğur, Muhammed Fatih Erbay, Hilal Güngör, Şefika İlknur Kökçü Karadağ, Aslı Berivan Topçak, Deniz Özçeker

**Affiliations:** ^1^ Department of Pediatric Allergy and Immunology Prof. Dr. Cemil Taşcıoğlu City Hospital Istanbul Turkey

**Keywords:** allergy, azithromycin, clarithromycin, cross reaction, drug allergy, intradermal test

## Abstract

**Background:**

Macrolide antibiotics are generally considered safe in children, but allergic reactions can still occur. This study aims to evaluate the sensitivity and specificity of intradermal test (IDT) used to detect macrolide allergy in pediatric patients, investigate the rate of cross‐reactivity between clarithromycin and azithromycin, and identify factors influencing the development of macrolide allergy.

**Methods:**

A total of 102 patients with suspected clarithromycin and azithromycin allergy were included in the study. Characteristics of the reactions and results of skin and drug oral provocation test (OPT) were recorded.

**Results:**

Clarithromycin was confirmed as the culprit drug in 8 (9%) of 88 patients, and azithromycin in 1 (7.1%) of 14 patients. The cross‐reactivity between clarithromycin and azithromycin determined 11.1%. In patients with immediate‐type reactions, IDT performed at a concentration of 0.05 mg/mL demonstrated a sensitivity of 33.3% (95% CI: 0.0%–66.7%) and a specificity of 92.7% (95% CI: 82.9%–100%). When a higher concentration of 0.5 mg/mL was used, sensitivity increased to 100% (95% CI: 0.0%–100%), while specificity decreased to 78.9% (95% CI: 65.8%–92.1%) respectively. According to univariate logistic regression analysis, patients with a history of previous non‐macrolide drug allergy (*p*: 0.003, Odds Ratio [OR]: 41, 95% CI: 3.6–456.5) and family history of drug allergy (*p* = 0.026, OR: 5.2, 95% CI: 1.2–22.5) were at a significantly higher risk of developing macrolide allergy.

**Conclusions:**

Although IDT at a concentration of 0.05 mg/mL showed higher specificity (92.7%) compared to 0.5 mg/mL (78.9%), given its limited sensitivity, OPT is still required to confirm the diagnosis.

## 1 Introduction

Macrolides, due to their broad antimicrobial spectrum, are among the first‐line antibiotics in patients with beta‐lactam allergy and represent a safe alternative for the treatment of various infections. Clarithromycin and azithromycin are the most commonly used agents within this group [1].

The low frequency of allergic reactions due to macrolides (0.4%–3%) may cause such reactions to be overlooked [2].

These reactions can range from mild skin rashes and symptoms such as urticaria and angioedema to, rarely, serious systemic reactions such as anaphylaxis [3, 4].

Skin prick tests (SPT), intradermal test (IDT), and patch tests are commonly utilized in diagnosing macrolide allergies; however, the sensitivity and specificity of these methods remain insufficiently documented in the literature. Studies investigating the effectiveness of IDT in macrolide allergy have yielded inconsistent findings [1, 5, 6].

Certain studies propose that IDT could play a role in diagnosing macrolide allergies, whereas others fail to provide adequate evidence supporting its reliability. These discrepancies underline the need for further research to clarify the diagnostic value of IDT in macrolide allergy [7, 8].

Although oral provocation tests (OPT) are considered the gold standard for the diagnosis of macrolide allergy, they are not always applicable in children with suspected allergy; in some clinical situations they are contraindicated. This makes the development of reliable skin tests even more important [9, 10].

The common structural features of macrolides may cause them to be similarly recognized by the immune system and this may increase the risk of cross‐reactions through IgE‐mediated or cellular immunity [9, 11]. Although cross‐reactions between clarithromycin and azithromycin have been rarely reported, the clinical significance of these reactions has not yet been fully elucidated. This uncertainty necessitates further research for patient safety and appropriate treatment [12]. It is clinically important to evaluate the reliability of diagnostic tests for macrolide allergy and to identify possible cross‐reactions.

Our aim was to determine the specificity and sensitivity of IDT for macrolide allergy in pediatric patients and to determine the rate of cross‐reactivity between clarithromycin and azithromycin.

## 2 Methods

### 2.1 Patient Group

The study included pediatric patients who were referred to our pediatric allergy outpatient clinic due to suspected macrolide allergy. Our study was designed retrospectively. Patients aged 2–18 years who underwent SPT, IDT, drug patch test, and/or OPT against macrolides between 2017 and 2024 were included. Patients without diagnostic tests, patients older than 18 years of age, patients with uncontrolled asthma, hypertension, cardiac diseases and patients who refused re‐exposure, patients with a history of severe reactions such as Stevens–Johnson syndrome, toxic epidermal necrolysis, drug reactions associated with eosinophilia and systemic symptoms, acute generalized exanthematous pustulosis, vasculitis, hepatitis and nephritis were excluded because drug OPT are not recommended in such cases.

Demographic characteristics of the patients included in the study (age, gender), presence of symptoms after macrolide drug intake (anaphylaxis, angioedema, urticaria, maculopapular rash, erythema, fixed drug eruption), the day and time of the reaction after drug intake, and the type of macrolide drug (clarithromycin or azithromycin), clinical and laboratory findings (hemogram, total IgE, eosinophil values), SPT, IDT, and drug OPT results were recorded from the patient files.

Reactions occurring within the first 1 h were classified as immediate type and reactions occurring after 6 h were classified as delayed type. Reactions occurring between 1 and 6 h were evaluated as immediate or delayed type considering both the clinical features and the time of occurrence of the reaction [13]. SPT or IDT were performed in patients with a history of immediate reactions to macrolides; patch tests and delayedreadings of IDT were performed in patients with a history of delayed reactions.

### 2.2 Skin Tests

SPT and IDT were performed to investigate macrolide allergy. Following the resolution of the rash/reaction to the suspected antibiotic, a waiting period of 6 weeks was observed before proceeding with further evaluations. Antihistamines were discontinued for 7 days, oral corticosteroids for 1 month, and topical steroids for 14 days prior to SPT. The skin test concentrations used in this study were selected based on previously published studies that identified nonirritant doses to ensure reliable testing and minimize false‐positive irritant reactions [7]. SPT was performed first. Clarithromycin was prepared at a concentration of 50 mg/mL and used for the SPT. The development of wheal at least 3 mm larger than the negative control within 15–20 min after the SPT was considered a positive reaction. If the SPT with clarithromycin was negative, IDT was performed. The IDT was performed at a concentration of 1:1000 (0.05 mg/mL) and if this dilution was negative, the concentration was increased to 1:100 (0.5 mg/mL) at 20 min intervals and the results were observed for 20 min (The test results are summarized in Table 1). Histamine (10 mg/mL) was used as a positive control and 0.9% NaCl as a negative control. An increase in the initial wheal diameter of more than 3 mm and the development of redness within 15–20 min was considered a positive result. Delayed readings were performed at 24, 48, and 72 h after intradermal testing to evaluate delayed‐type hypersensitivity reactions. The term “delayed reading of IDT” refers to the re‐evaluation of the test site at 24, 48, and 72 h after injection, in order to detect delayed‐type hypersensitivity responses [13].

**TABLE 1:** Evaluation of demographic, clinical, and test findings in suspected macrolide allergy.

Age (months) mean ± SD (min–max)		72 ± 45.8 (12–250) *n* (%)
Gender	Female	55 (53.9)
	Male	47 (46.1)
Drug	Azithromycin	14 (13.7)
	Clarithromycin	88 (86.3)
Clinical symptoms	Urticaria	55 (53.9)
	Angioedema	10 (9.8)
	Urticaria‐angioedema	1 (1.0)
	Anaphylaxis	5 (4.9)
	Maculopapular rash	31 (30.4)
Reaction timing	First 1 h	28 (27.5)
	1–6 h	47 (46.1)
	> 6 h	27 (26.5)
Temporal onset of reaction	Delayed reaction	32 (31.4)
	Immediate reaction	70 (68.6)
Time between reaction and test (months) mean ± SD (min–max)		3.7 ± 1.4 (2–8)
IgE mean ± SD (min–max)		169.1 ± 251.8 (2–1316)
SPT result	Negative	81 (98.7)
	Positive	1 (1.3)
IDT result for clarithromycin	Negative	48 (59.2)
	Positive at 0.5 mg/mL	28 (34.5)
	Positive at 0.05 mg/mL	5 (6.1)
Patch test	Negative	26 (100)
IDT delayed reading result for clarithromycin	Negative	81 (100)
Drug OPT result	Negative	83 (81.4)
	**Positive**	**9 (8.8)**
	Not performed due to anaphylaxis	5 (4.9)
	Not performed due to patient refusal	4 (3.9)
	Not performed due to positive SPT	1 (1.0)
Reactions caused by OPT	Urticaria	7 (77.8)
	Maculopapular rash	2 (22.2)
Clarithromycin OPT result in patients with positive azithromycin OPT	Negative	1 (100)
Azithromycin OPT result in patients with positive clarithromycin OPT	Negative	8 (88.9)
	Positive	1 (11.1)
Additional allergic diseases	None	35 (36.4)
	Asthma	13 (12.7)
	Allergic rhinitis	26 (25.5)
	Asthma + allergic rhinitis	9 (8.8)
	Atopic dermatitis	3 (2.9)
	Food allergy	14 (13.7)
	Previous non‐macrolide DHR (confirmed by OPT)	5 (4.9)
	Ceftriaxone	2 (2.0)
	Amoxicillin‐clavulanic acid	3 (2.9)
Family history of drug allergy	None	84 (82.4)
	Present	18 (17.6)

*Note:* Data that were statistically significant are highlighted in bold.

Abbreviations: DHR, Drug Hypersensitivity Reaction; IDT, Intradermal Test; OPT, Oral Provocation Test; SD, Standard Deviation; SPT, Skin Prick Test.

Due to the absence of a sterile injectable form of azithromycin, which is necessary for safe and reliable SPT and IDT, these procedures were not conducted. Instead, azithromycin hypersensitivity was assessed through patch testing and OPT.

### 2.3 Patch Test

Patients with delayed‐type reactions who consented to patch testing were subjected to patch testing using standardized clarithromycin and azithromycin patch test preparations (Chemotechnique Diagnostics, Sweden) using Finn Chambers on the upper back. Test results were evaluated after 48 and 72 h.

### 2.4 Oral Provocation Tests

For confirmation of macrolide allergy, OPT were performed in the hospital. OPT of suspected drugs were performed according to European Network for Drug Allergy guidelines [14]. The suspected drug was administered in increasing doses every 30 min until reaching a total dose adjusted for the patient’s age and weight. The total daily dose of clarithromycin was set at 15 mg/kg. The provocation test was planned to begin with 1/10 of a single dose, followed by increases to half and then the full dose, with 30‐min intervals between each step, completing the process in three stages. For azithromycin, the total dose was set at 5 mg/kg/day and a similar dose escalation protocol was applied. If any objective symptoms such as urticaria, angioedema, maculopapular rash, cough, wheezing, dyspnea, or vomiting were observed during the test, the result was considered positive and drug administration was discontinued [14].

If a reaction occurred as a result of OPT, the severity, time of onset and other accompanying symptoms were recorded. Patients with negative test results were advised to use the drug at home at therapeutic doses for 5 days to evaluate delayed‐type reactions.

In certain cases, SPT, IDT, or OPT were not performed due to specific clinical circumstances—such as patient refusal, a history of high‐risk anaphylaxis, or a previously positive SPT result that was deemed sufficient to establish the diagnosis without further testing.

### 2.5 Ethics Statement

Ethical approval for this study was obtained from the Ethics Committee of our hospital in 16.09.2024 (Approval No: 17).

### 2.6 Statistical Analysis

Data were analyzed with SPSS (Statistical Package for Social Sciences) version 23.0 software. Continuous variables were expressed as mean and standard deviation, while categorical variables were presented as frequency and percentage. The normality of the distribution was evaluated by Kolmogorov–Smirnov and Shapiro–Wilk tests. Normally distributed data were analyzed with parametric tests and non‐normally distributed data were analyzed with non‐parametric tests. *p* < 0.05 was accepted as the limit of statistical significance. Sensitivity and specificity rates were calculated and given with 95% confidence intervals.

## 3 Results

### 3.1 Demographic and Clinical Characteristics

A total of 102 patients were included in the study, comprising 55 females (53.9%) and 47 males (46.1%). The mean age of the cohort was 72 months. Isolated urticaria was the most frequently observed diagnosis, comprising 53.9% of cases, followed by maculopapular rash with (30.4%) and angioedema (9.8%). The suspected macrolide was clarithromycin in 88 (86.3%) patients and azithromycin in 14 (13.7%) patients. Seventy patients (68.6%) presented with immediate reactions and 32 (31.4%) with delayed reactions. Demographic information of the patients is given in Table 1.

### 3.2 Diagnostic Evaluation of Clarithromycin Allergy

A total of 88 patients were suspected of having clarithromycin allergy in the study. A total of 58 patients presented with suspected immediate‐type hypersensitivity reactions attributed to clarithromycin, whereas 30 patients were evaluated for delayed‐type reactions following clarithromycin exposure.

#### 3.2.1 Immediate‐Type Hypersensitivity Reactions (*n* = 58)

Among the 58 patients presenting with suspected immediate‐type hypersensitivity reactions to clarithromycin, two patients underwent direct OPT both of which yielded negative results. The remaining 56 patients were first evaluated with a SPT. One patient demonstrated a positive SPT result; this patient had presented with angioedema, and OPT was not performed.

In the remaining 55 patients with negative SPT results, IDT was initiated at a concentration of 1/1000 (0.05 mg/mL). IDT was positive in 5 of these patients. Among them, two had a positive OPT result, while the remaining three had negative OPT outcomes.

**FIGURE 1:**
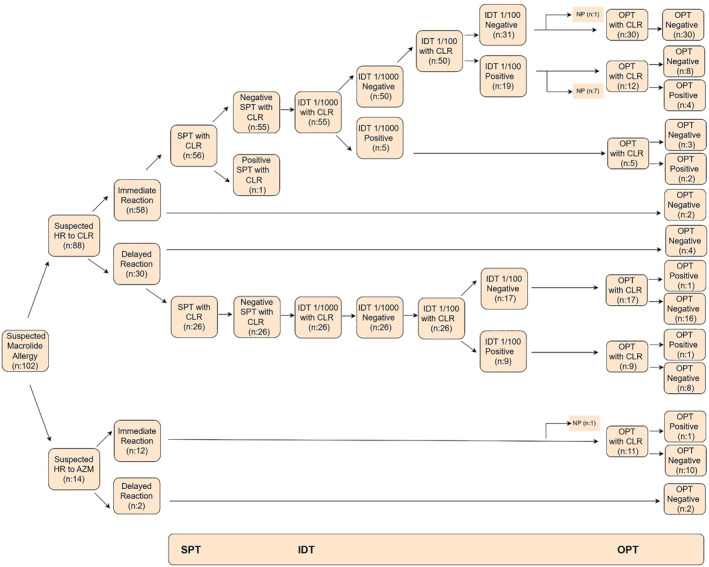
Graded diagnostic evaluation of 102 patients with suspected macrolide hypersensitivity. A total of 102 patients with suspected macrolide allergy were evaluated. Of these, 88 were examined with suspected CLR and 14 with suspected AZM hypersensitivity. Of the patients with CLR reactions, 58 had a history of immediate and 30 had a history of delayed type reactions. SPT was performed in 56 of the patients with immediate reactions; SPT positivity was observed in only 1 patient. IDT at 1/1000 dilution was performed in all 55 SPT negative patients and positivity was detected in 5 patients. IDT at 1/100 concentration was performed in 50 patients who were IDT negative and positivity was detected in 19 patients. Positive results were obtained in a total of 6 patients with immediate reaction. A total of 26 patients with delayed reaction were evaluated with SPT and IDT; no positivity was observed in SPT. Of the 26 patients with negative IDT 1/1000, 9 were positive on IDT 1/100. Among the patients with delayed reactions, a total of 2 patients were positive on OPT. Of the 14 patients with suspected azithromycin reaction, 12 had immediate type reaction and 2 had delayed type reaction; OPT was negative in patients with delayed type reaction and OPT was performed in 11 patients with immediate type reaction and positivity was detected in 1 patient. AZM, Azithromycin; CLR, Clarithromycin; HR, Hypersensitivity Reaction; IDT, Intradermal Test; NP, Not performed; OPT, Oral Provocation Test; SPT, Skin Prick Test.

Subsequently, the remaining 50 patients underwent IDT at a higher concentration of 1/100 (0.5 mg/mL). Nineteen of these patients tested positive. Of those 19 patients, OPT was not performed in 7, while 4 had a positive OPT and 8 had a negative OPT. The remaining 31 patients had negative IDT results at the 1/100 concentration. Among these, OPT could not be performed in one patient, and the remaining 30 patients had negative OPT results. Diagnostic test results are summarized in Figure 1.

The diagnostic performance of IDT was evaluated in a subset of 47 patients with suspected immediate‐type reactions to clarithromycin who underwent both IDT and OPT. Sensitivity, specificity, and predictive values of IDT were calculated based on this group. The detailed results are presented in Table 2.

**TABLE 2:** Results of IDT and OPT in patients with immediate reactions to clarithromycin.

	IDT result	OPT negative	OPT positive	Total
A. Comparison of IDT (0.05 mg/mL) and OPT results				
Immediate reactions	Negative	38	4	42
	Positive	3	2	5
	Total	41	6	47
B. Comparison of IDT (0.5 mg/mL) and OPT results				
Immediate reactions	Negative	30	0	30
	Positive	8	4	12
	Total	38	4	42

Abbreviations: IDT, Intradermal Test; OPT, Oral Provocation Test.

At a concentration of 0.05 mg/mL, IDT demonstrated a sensitivity of 33.3% (95% CI: 0.0%–66.7%) and a specificity of 92.7% (95% CI: 82.9%–100%). The positive predictive value (PPV) was 40.0% (95% CI: 0.0%–80%), and the negative predictive value (NPV) was 90.5% (95% CI: 81.0%–97.6%).

At the higher concentration of 0.5 mg/mL, the sensitivity increased to 100% (95% CI: 0.0%–100%), while the specificity decreased to 78.9% (95% CI: 65.8%–92.1%). The PPV was 33.3% (95% CI: 8.3%–58.3%), and the NPV was 100% (95% CI: 0.0%–100%).

#### 3.2.2 Delayed‐Type Hypersensitivity Reactions (*n* = 30)

Among the 30 patients presenting with suspected delayed‐type hypersensitivity reactions to clarithromycin, four underwent direct OPT, all of which yielded negative results. The remaining 26 patients were initially tested with a SPT, and all results were negative.

Subsequently, all 26 patients underwent IDT at a 1/1000 (0.05 mg/mL) concentration, which also yielded negative results in all cases. As the next step, IDT was repeated at a higher concentration of 1/100 (0.5 mg/mL). Of these, 9 patients (34.6%) had a positive IDT result. Among them, only one patient had a positive OPT, while the remaining eight had negative OPT outcomes. The other 17 patients had negative IDT results at the 1/100 concentration. Of these, one patient had a positive OPT, and 16 had negative results (Table 3).

**TABLE 3:** Results of IDT and OPT in patients with delayed reactions to clarithromycin.

	IDT result	OPT negative	OPT positive	Total
A. Comparison of IDT (0.05 mg/mL) and OPT results				
Delayed reactions (IDT immediate reading)	Negative	24	2	26
	Positive	0	0	0
	Total	24	2	26
B. Comparison of IDT (0.5 mg/mL) and OPT results				
Delayed reactions (IDT immediate reading)	Negative	16	1	17
	Positive	8	1	9
	Total	24	2	26
C. Delayed IDT and OPT results in delayed reactions				
Delayed reactions (IDT delayed reading)	Negative	24	2	26
	Positive	0	0	0
	Total	24	2	26

Abbreviations: IDT, Intradermal Test; OPT, Oral Provocation Test.

All patients had negative delayed IDT readings at 24, 48, and 72 h.

In patients with suspected delayed‐type hypersensitivity to clarithromycin, the diagnostic performance of IDT was assessed based on both immediate and delayed readings, as some patients exhibited positive results in the immediate readings despite a clinical suspicion of delayed reactions.

Delayed readings for both concentrations were negative in all patients, resulting in a sensitivity of 0.0% and a specificity of 100% (95% CI: 0.0%–100%), with an NPV of 92.3% (95% CI: 80.8%–100%). Again, due to the absence of any positive delayed responses, PPV could not be calculated. There were 20 patients who underwent both patch testing and OPT with clarithromycin. In the patch test with clarithromycin, all 20 patients had negative results; however, 2 of these patients had a positive OPT.

### 3.3 Diagnostic Evaluation of Azithromycin Allergy

A total of 14 patients were evaluated for suspected azithromycin allergy. Two patients had a history of delayed‐type reactions, while 12 reported immediate‐type reactions. OPT were negative in both patients with delayed‐type reactions. Among the 12 patients with immediate‐type reactions, one had a positive OPT result, one declined the test, and the remaining 10 had negative OPT results.

All five patients who underwent patch testing with azithromycin had negative results, and none of them showed a positive OPT. One patient with a negative patch test did not undergo OPT. In conclusion, no positive results were observed in patch testing throughout the study.

### 3.4 Oral Provocation Test Outcomes and Cross‐Reactivity in Patients With Suspected Macrolide Allergy

A total of 92 patients underwent OPT to evaluate allergic reactions to the suspected macrolide. Among them, 9 patients who had a positive OPT result for the suspected drug subsequently underwent an additional OPT with another macrolide derivative to assess potential cross‐reactivity. OPT was not performed in 5 patients due to a history of severe anaphylaxis. Additionally, 4 patients declined testing, and 1 patient was not tested because of a positive SPT. The OPT yielded positive results in 9 patients. In this study, clarithromycin was identified as the culprit drug in 8 of 88 patients with suspected clarithromycin allergy (9%), while azithromycin was confirmed as the causative agent in only 1 of 14 patients (7.1%) with suspected azithromycin allergy.

The clinical characteristics of the 9 patients with confirmed macrolide hypersensitivity by OPT are summarized below:

Patient 1 was a 35‐month‐old male who developed immediate‐onset urticaria (≤ 1 h) after clarithromycin exposure. IDT was positive at 0.05 mg/mL, and urticaria was reproduced during OPT.

Patient 2 was a 133‐month‐old female who developed urticaria 1–6 h after azithromycin administration, and the reaction recurred during OPT.

Patient 3 was a 171‐month‐old female who developed urticaria within 1 h of clarithromycin intake. IDT at 0.05 mg/mL was positive, and urticaria was reproduced during both clarithromycin and azithromycin OPTs.

Patient 4 was a 50‐month‐old female who developed urticaria 1–6 h after clarithromycin exposure. IDT was positive at 0.5 mg/mL, and the same reaction occurred during OPT.

Patient 5 was a 168‐month‐old male who developed urticaria within 1 h of clarithromycin administration. IDT was positive at 0.5 mg/mL, and the reaction recurred during OPT.

Patient 6 was an 80‐month‐old male who developed a maculopapular eruption more than 24 h after clarithromycin use. IDT was positive at 0.5 mg/mL, and the same rash reappeared 12 h after OPT.

Similarly, Patient 7 was a 26‐month‐old male who developed delayed‐onset MPE following clarithromycin exposure. Both SPT and IDT were negative, but the clinical eruption was reproduced 8 h after OPT.

Patient 8 was a 62‐month‐old female who developed urticaria 1–6 h after clarithromycin intake. IDT was positive at 0.5 mg/mL, and urticaria recurred during OPT.

Finally, Patient 9 was a 120‐month‐old female who developed immediate onset urticaria (≤ 1 h) following clarithromycin administration. IDT was positive at 0.5 mg/mL, and the reaction was reproduced during OPT.

**FIGURE 2:**
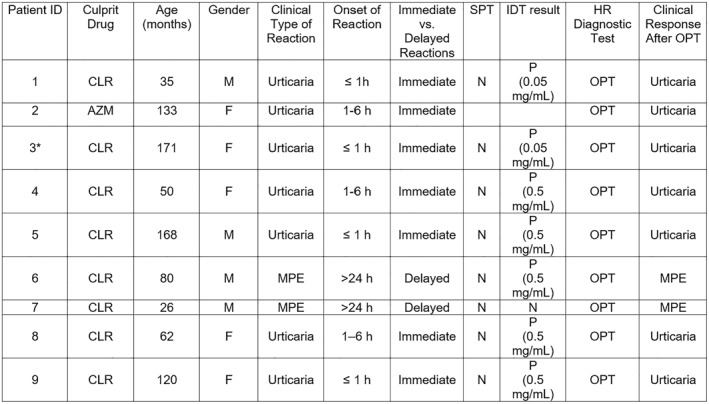
Characteristics of patients with positive OPT results for clarithromycin and azithromycin. This figure summarizes the demographic and clinical characteristics of the nine patients who had positive OPT results for macrolides. It includes information on the culprit drug, age, sex, type, and timing of clinical reaction, SPT and IDT results, as well as the clinical manifestations observed following OPT. AZM, Azithromycin; CLR, Clarithromycin; F, Female; HR, Hypersensitivity Reaction; IDT, Intradermal Test; IMPT, Intramuscular provocation test; M, Male; MPE, Maculopapular Eruption; N, Negative; OPT, Oral Provocation Test; P, Positive; SPT, Skin Prick Test. * Patient no. 3 exhibited a positive oral provocation test result to both azithromycin and clarithromycin, indicating a cross‐reactive hypersensitivity between the two macrolides.

Among the 8 patients with a positive OPT for clarithromycin, one also had a positive OPT result with azithromycin, resulting in a cross‐reactivity rate of 11.1% between clarithromycin and azithromycin. In contrast, clarithromycin OPT was negative in the patient with a positive azithromycin OPT. The detailed characteristics of all patients with a positive OPT are presented in Figure 2.

### 3.5 Factors Associated With Positive Oral Provocation Test Results in Macrolide Allergy

In patients with suspected macrolide allergy, 4 patients with previous hypersensitivity reactions to non‐macrolide drugs, confirmed by OPT, were identified. Two of these patients had hypersensitivity to amoxicillin‐clavulanic acid, and two to ceftriaxone. OPT results were positive in all four cases. Macrolide allergy was also confirmed in three of these patients. In patients with a positive macrolide OPT result, the rates of previous hypersensitivity reactions to non‐macrolide drugs (*p* = 0.003), comorbid allergic diseases (*p* = 0.044), and a family history of drug allergy (*p* = 0.036) were significantly higher compared to those with a negative result (Table 4).

**TABLE 4:** Evaluation of OPT results in suspected macrolide allergy.

*N* = 92		Drug OPT result				*p*
		Negative		Positive		
		*n*	%	*n*	%	
Gender	Male	38	45.8	4	44.4	1.000
	Female	45	54.2	5	55.6	
Age (months) mean ± SD		75.5 ± 44.3		93.9 ± 55.7		0.327
Drug	Azithromycin	12	14.5	1	11.1	1.000
	Clarithromycin	71	85.5	8	88.9	
Clinical symptoms	Urticaria	46	55.4	7	77.8	0.475
	Angioedema	8	9.6	0	0.0	
	Maculopapular rash	29	34.9	2	22.2	
Reaction timing	First 1 h	17	20.5	4	44.4	0.340
	1–6 h	41	49.4	3	33.3	
	> 6 h	25	30.1	2	22.2	
Temporal onset of reaction	Delayed reaction	30	36.1	2	22.2	0.488
	Immediate reaction	53	63.9	7	77.8	
Time between reaction and test (months) mean ± SD (min–max)		3.7 ± 1.5		3.6 ± 1.0		0.819
IgE mean ± SD		178.7 ± 263.3		95.4 ± 126.2		0.517
Additional allergic diseases	None	33	39.8	0	0.0	0.024
	Yes	50	60.2	9	100	
	Asthma	8	9.6	2	22.2	0.252
	Allergic rhinitis	20	24.1	3	33.3	0.686
	Asthma + allergic rhinitis	8	9.6	1	11.1	1.000
	Atopic dermatitis	3	3.6	0	0.0	1.000
	Food allergy	10	12	3	33.3	0.112
	Previous non‐macrolide DHR	1	1.2	3	33.3	0.003
Family history of drug allergy	None	72	86.7	5	55.6	0.036
	Present	11	13.3	4	44.4	

*Note:* Data that were statistically significant are highlighted in red color.

Abbreviations: DHR, Drug Hypersensitivity Reaction; IDT, Intradermal Test; OPT, Oral Provocation Test; SD, Standard Deviation.

According to univariate logistic regression analysis, patients with a previous drug hypersensitivity reaction were at a significantly higher risk of developing macrolide allergy (*p*: 0.003, OR: 41, 95% CI: 3.6–456.5). In addition, family history of drug allergy was found to be a significant risk factor for macrolide allergy (*p* = 0.026, OR: 5.2, 95% CI: 1.2–22.5).

In multivariable analysis, no variable showed a significant association with macrolide allergy.

## 4 Discussion

Macrolides are among the antibiotics with relatively low rates of allergic reactions in the treatment of bacterial infections. However, the lack of standardized diagnostic tests in macrolide allergy complicates the diagnosis and treatment process, and this may lead to unnecessary drug restrictions and forced patients to turn to less appropriate alternative treatment options.

In our study, the rate of confirmed macrolide allergy among patients with suspected hypersensitivity was 8.8%. Similarly, previous studies have reported low confirmation rates after OPT, such as 9.8% and 6%. The low rate of confirmed macrolide allergy may be due to infection‐related rashes being misclassified as drug allergy or the involvement of non‐immunological mechanisms [7, 15]. In our study, the confirmed allergy rate to clarithromycin was 9.1%, and the rate of allergy to azithromycin was 1.1%. The findings of our study showed that the rate of confirmed allergy to azithromycin was lower compared to clarithromycin. This difference may be due to lower exposure of patients to azithromycin due to its lower use in our country.

However, studies comparing the allergenicity of clarithromycin and azithromycin have yielded conflicting results [6, 16].

Skin tests for macrolide antibiotics have not yet been validated because the results of studies are limited and contradictory. Total skin test positivity rate in clinical studies has been from 28% to 43% among adults with suspected macrolide hypersensitivity [4].

In our study, IDT performed with a concentration of 0.05 mg/mL demonstrated a specificity of 92.7% in immediate reactions, whereas a higher concentration of 0.5 mg/mL resulted in a lower specificity of 78.9%. These findings suggest that lower, non‐irritant concentrations may provide more reliable diagnostic accuracy by reducing false‐positive results.

In a study in healthy adult subjects, Broz et al. suggested that a 1:1000 dilution of a 50 mg/mL commercial formulation of clarithromycin was the highest safe concentration that did not cause skin irritation [17]. In a study evaluating adult patients who reported that skin tests were not reliable in predicting macrolide hypersensitivity, the specificity of all skin tests was 85%, NPV 50%, PPV 0%, false positive rate 3% and false negative rate 17% [8]. Although IDT was performed across a range of concentrations (0.01–10 mg/mL), sensitivity and specificity were not reported for each concentration individually, limiting interpretation.

In a retrospective study of adult patients by Seitz et al., patients with suspected erythromycin, clarithromycin, roxithromycin, and azithromycin allergy were evaluated and positive IDT results were obtained in only 1 patient among 125 patients [18]. The IDT concentration of 0.01 mg/mL used in this study is a lower concentration compared to the starting concentration of 0.05 mg/mL in our study. Although lower concentrations such as 0.01 mg/mL are associated with increased safety and reduced risk of systemic reactions, they may lead to reduced sensitivity. Conversely, concentrations such as 0.05 mg/mL may improve sensitivity but potentially lower specificity. Therefore, the diagnostic performance of IDT may vary depending on the concentration used, and this should be considered when interpreting results across different studies. In our study, IDT was initiated with 0.05 mg/mL and the positive results were confirmed by OPT, whereas in the study by Seitz et al., only 0.01 mg/mL was used and no diagnostic performance data were provided, resulting in a low rate of positivity.

However, in some studies, IDT have been found to have good specificity and sensitivity and to be useful in allergic evaluation. In the study by Mori et al., when the results of IDT were compared with the results of single‐blind placebo‐controlled OPT, the sensitivity and specificity of IDT were 75% and 90%, respectively. The NPV was high (98%), while the PPV was low (33%) [7]. This has been attributed to the low prevalence (6%) of clarithromycin allergy in the study population. Three of the four patients with positive OPT results also had immediate positive IDT responses at 0.5 mg/mL, and 54 of the 60 patients with negative OPT results also had negative IDT results, indicating high specificity. According to the current results, the 0.5 mg/mL dilution was found to be the non‐irritant threshold concentration for IDT with clarithromycin. In this study, a 5 mg/mL concentration was reported to elicit a positive response in 39% of children in the control group, indicating that this dose may have an irritant effect. Similarly, a non‐irritant dose was used in our study as well. In this study, it was suggested that the use of IDT would reduce the need for OPT.

In a previous study, the sensitivity, specificity, PPV, and NPV of clarithromycin IDT were 50%, 69.6%, 12.5%, and 92.1%, respectively. In this study, a dose of 5 mg/mL was used during IDT [19]. The relatively low specificity reported in the previous study may be attributed to the use of a higher concentration (5 mg/mL), which could potentially lead to irritant reactions. In contrast, in our study, non‐irritant doses defined by Mori et al. in healthy children were used, and high specificity was achieved at the concentration of 0.05 mg/mL [7].

In one study, only 2 of 21 patients with suspected immediate‐type hypersensitivity to clarithromycin had a positive skin test (1 with a positive SPT result and 1 with a positive IDT result), but since OPT was not performed in these two patients, PPV could not be calculated. On the other hand, all OPT results of 19 patients with negative IDT were negative. In the same study, OPT was positive in 2 of 32 patients with a history of delayed‐type reaction and negative IDT while it was negative in 30 patients. In this study, the NPV of IDT for clarithromycin was 100% in immediate reactions and 94% in delayed reactions. In our study, the diagnostic performance of IDT according to different concentrations was evaluated in detail; the high specificity of the 0.05 mg/mL dose and the confirmation of positive results with OPT provided more reliable data compared to previous studies [16].

In the study by Cavkaytar et al., 20 patients underwent IDT and OPT with clarithromycin. IDT was positive in a total of 9 patients, including 2 patients at a dilution of 1:100,000, 2 patients at 1:10,000 and 5 patients at 1:1000. However, none of these patients developed a clinical reaction during OPT. In contrast, two patients with negative IDT developed urticaria during OPT. In the study, 9 out of 20 patients (45.5%) had positive skin tests, which is higher than the rates reported in the literature and this may be explained by the possibility of false positivity due to the practitioner. Furthermore, this study did not test with a concentration of 1/100, which is recommended in previous literature as a non‐irritant dose. If this concentration had been included in the test protocol, it would have been possible to detect the two OPT positive patients by skin tests. These findings indicate that uncertainties remain regarding the sensitivity and specificity of IDT with clarithromycin, especially in pediatric patients [20].

Although our algorithm recommends performing an OPT to confirm a diagnosis of macrolide allergy in cases where the IDT is negative, performing an OPT in routine pediatric practice presents certain practical and clinical difficulties. Clinicians may not always opt for OPT due to concerns from families about potential reactions, the drug no longer being considered essential for treatment, or the initial reaction presenting with a severe clinical picture. Additionally, the therapeutic necessity of the drug and the antimicrobial usage spectrum directly influence this decision‐making process. In our study, IDT negativity was not always sufficient to rule out allergy; in some patients, OPT was positive despite IDT negativity. Therefore, while OPT is considered diagnostically necessary, its applicability should be evaluated on a case‐by‐case basis, considering the risk‐benefit balance for each patient.

The structure of macrolides consists of a large lactone ring ranging in size from 12 to 18 atoms. Current data are insufficient to understand cross‐sensitization between macrolide derivatives [1]. Cross‐reaction has also been reported between azithromycin and clarithromycin although they contain different numbers of carbon atoms [12]. In a single‐blind placebo‐controlled study in adult patients, cross‐reactivity between four different macrolide antibiotic groups (clarithromycin, azithromycin, dirithromycin, spiramycin) was 20%. In this study, allergy to azithromycin was observed in 2 of 20 clarithromycin allergic patients and allergy to clarithromycin in 1 of 2 azithromycin allergic patients [8].

In our study, the rate of cross‐reaction between clarithromycin and azithromycin was 11%. However, since this finding is based on only one case and the sample size is limited, the possibility of a random allergy cannot be ruled out. This observation should be interpreted with caution and confirmed by future studies. In another study conducted in our country, cross‐reaction with clarithromycin was confirmed in 33.3% of patients with azithromycin allergy [19]. In a study conducted by Mori et al., cross‐reactivity between macrolide antibiotics with differing ring structures was demonstrated. In this case series, one pediatric patient developed anaphylaxis to both clarithromycin and azithromycin, and all cases showed positive skin test results, indicating a shared immunologic response [12].

Pereira et al. administered IDT to 46 patients suspected of having macrolide allergy and obtained positive results in 10.9% (*n* = 5) of cases. However, none of these patients accepted drug OPT to confirm the diagnosis. A total of 48 OPTs were administered to patients with negative IDTs; 25 of these were with clarithromycin, 22 with azithromycin, and 1 with erythromycin. The positive OPT rate was 6.25%, and all reactions were mild and limited to cutaneous symptoms. In two cases of cross‐reactivity, positive reactions to both azithromycin and clarithromycin were reported [21].

These findings support the notion that cross‐sensitization may occur even between structurally dissimilar macrolide agents. Although the number of patients showing cross‐reactivity in the studies was limited, these data provide an important contribution given the limited knowledge in the current literature.

In our study, the presence of confirmed previous drug allergy diagnosed by OPT was found to be a significant risk factor in patients with macrolide allergy in the univariate analysis. Multiple drug hypersensitivity is a condition that develops through T‐cell‐mediated mechanisms and is characterized by persistent reactions to structurally unrelated drugs; sustained T‐cell activity may lead to the development of new drug allergies [22]. In our study, confirmed previous drug allergy was detected with amoxicillin‐clavulonic acid and ceftriaxone. In the study by Suleyman et al., switching to clarithromycin treatment after amoxicillin‐clavulanic acid (sequential use) and confirmed beta lactam allergy were found to be important risk factors for clarithromycin hypersensitivity [23]. Our findings highlight the importance of careful evaluation in patients with a history of drug allergy.

In our study, family history of drug allergy was found to be a significant risk factor in patients with macrolide allergy. In the literature, it has been reported that 10% of family members of patients with drug allergy develop drug allergy [24]. Although genetic predisposition may contribute to drug allergy and justify caution in those with a family history, the supporting evidence remains limited and controversially discussed [25].

However, when multivariate logistic regression was performed, none of the variables, including previous drug allergy and family history of drug allergy, remained statistically significant. This may be due to the limited number of confirmed cases of macrolide allergy in our study, and this limitation reduces the statistical power of the model. Therefore, findings that are significant in univariate analysis should be interpreted with caution.

The limitation of our study is that it was conducted in a single center and the results cannot be generalized. The strength of our study is that a large number of patients were diagnosed by OPT in an area with limited data in the literature. To our knowledge, this study is one of the most comprehensive studies on macrolide allergy in children and provides comprehensive data on diagnostic approaches to this group of antibiotics, which are commonly prescribed in clinical practice [26]. Another strength is that patients were evaluated separately according to immediate and delayed type reactions and sensitivity and specificity values for each IDT concentration were reported in detail.

## 5 Conclusion

Although IDT at a concentration of 0.05 mg/mL demonstrated higher specificity (92.7%) compared to 0.5 mg/mL (78.9%), its limited sensitivity necessitates OPT to confirm the diagnosis.

## Author Contributions

**Güler Yıldırım:** writing – original draft, conceptualization, investigation, funding acquisition, methodology, data curation, writing – review and editing. **Merve Karaca Şahin:** data curation, software. **Nilay Çalışkan:** data curation, software. **Hamit Boloğur:** data curation, software. **Muhammed Fatih Erbay:** data curation, software. **Hilal Güngör:** data curation, software, validation. **Şefika İlknur Kökçü Karadağ:** data curation, writing – review and editing, visualization. **Asli Berivan Topçak:** data curation, software, formal analysis. **Deniz Özçeker:** writing – review and editing, conceptualization, supervision.

## Conflicts of Interest

The authors declare no conflicts of interest.

## Data Availability

The datasets generated during the current study are available from the corresponding author on reasonable request.
